# A Review of Recent Progress in Synthetic Polymer Surface Coatings for the Prevention of Biofilm Formation

**DOI:** 10.3390/molecules30132710

**Published:** 2025-06-24

**Authors:** Adrienne Shea, Matthew T. Bernards

**Affiliations:** Department of Chemical and Biological Engineering, University of Idaho, Moscow, ID 83844, USA; shea2697@vandals.uidaho.edu

**Keywords:** biofilm, biofouling, bacterial adhesion, hydrogels, polyampholytes, zwitterions, poly(oxazoline), PEG, nonfouling

## Abstract

Bacterial adhesion and the subsequent formation of biofilms and biofouling have significant economic and health impacts across all sectors. They are especially impactful in industrial corrosion, healthcare, food processing, agriculture, and waste and drinking water. Synthetic polymers that resist bacterial adhesion are adaptable to a wide range of applications in all of these fields. While there are many bacteria-resistant polymers, some of the best performing include polyethylene glycol (PEG), poly(oxazoline) (POZ), and zwitterionic polymers, with zwitterionic polymers showing the most promise with reductions in bacteria adhesion up to 99% over controls. This review summarizes the demonstrated bacterial resistance performance of these polymer coatings based on literature published over the last ten years. It also identifies the front runners for preventing bacterial adhesion while providing the critical next steps for widespread adoption of this technology.

## 1. Introduction

Every year significant resources are devoted to combating biofilms as an expansive challenge for human life and industry [1]. The estimated annual financial burden reported in 2019 was in excess of $5 trillion USD worldwide [1] with anti-microbial films reaching a global market of $4.28 billion USD in 2021 [2]. Reported financial costs imposed by biofilms do not include the costs for constant research and development which is necessary as bacteria become resistant to biocides and antimicrobials [3,4,5].

Traditionally, biofilms have been defined as bacterium contained within a three-dimensional, excreted extracellular polymeric matrix attached to a surface [5]. However, advancements in the scientific understanding of how biofilms form and function have expanded that definition to include not only surface-attached microbial aggregates, but also non-surface-attached aggregated bacteria [6]. In this review, references to biofilms are specific to surface-attached biofilms. Biofilms have a complex life cycle that leads to enhanced resistance to antimicrobial agents, tolerance to desiccation, shear stress, and protozoan grazing, and the increased capture of nutrients compared to free-living bacterial cells [4,6,7]. In addition to these benefits, surface-attached biofilms condition the surfaces they interact with by altering the physicochemical properties of the substratum surface, making repeat growth virtually impossible to prevent or eliminate [8,9]. Given the inherent challenges of treating biofilms with a reactive process, significant research efforts are dedicated towards the prevention of initial biofilm formation. Most prevention techniques utilize a surface modification or coating that impedes initial bacterial surface attachment, which in turn prevents surface-attached biofilm formation.

While the focus of this review is not on the impacts of biofouling, a few pertinent examples are provided to better frame its widespread impact. Biofouling in industrial systems not only causes the corrosion or degradation of surfaces and reductions in product quality, but also potentially exposes workers to aerosolized particles during processing or cleaning [10]. Any processes containing membranes are also significantly affected by biofilm formation; this biofilm then acts as a secondary membrane, imposing an increased pressure drop and reducing flux through the affected membrane [9]. Healthcare and the medical field are impacted in multiple ways, from dentistry and implants to chronic wound control, which have been the focus of many recent reviews [11,12,13,14,15,16,17,18]. Foodborne illnesses are a highly publicized result of biofilm formation in every stage of the food supply chain [19,20,21,22]. Agricultural irrigation systems are susceptible to biofouling, and a study in 2015 found that even the residual water remaining in the pipelines between irrigation events fosters significant bacterial growth [23]. Additionally, irrigation water and pipelines have been investigated for their role in disseminating antibiotic-resistant bacteria into the environment [23]. Biofilms have significant implications for wastewater and potable water, including corrosion and blockages of components, leading to mechanical failure within the systems [5,24,25,26,27,28]. Potable water systems contaminated with biofilm also cause at least 7 million incidents annually in the United States alone [6,29,30,31,32,33,34,35]. A common thread throughout the afflicted systems, excluding the complicated systems within the medical field, is the presence of pipelines and stagnation points that are susceptible to biofilm formation [5,9,10].

Across all sectors afflicted with the impacts of biofilm formation, the current mode of mitigation is treatment and control, generally including forms of UV/radiation treatment [36,37,38], antimicrobial biocides [3,39,40,41,42,43,44], biocidal-impregnated surfaces and coatings [45,46,47,48,49,50,51,52,53,54], signal-disrupting chemicals or enzymes [55,56,57,58,59], oxidants [60,61], or physical cleaning including aeration [62,63,64,65]. Each of these processes reduces bacterial loads within systems, but none reach total eradication. Mechanical scrubbing and other physical cleaning can also be impossible or impractical in confined spaces [66]. Biofilms inherently act as barriers for the bacteria contained within, which means the innermost layers of the biofilm are exposed to sublethal doses of antimicrobials, leading to acquired resistance [8]. In all these treatments, the inherent flaw is that even if 99% of the micro-organisms within the system are eliminated, the remainder will still recolonize the surface-conditioned interior of the system [67]. As an example, out of all waterborne pathogen outbreaks in the United States, the CDC reports that 54% of the contaminated systems were previously treated with disinfectants [29]. Therefore, the focus of the remainder of this review will be developing approaches for preventing the initial stages of bacterial adhesion to prevent biofilm formation from initiating.

## 2. Prevention

While there are many approaches to prevent bacterial adhesion, including surface hydrophobicity modifications by hydrophobic coatings or micropatterning [68,69,70,71,72,73] and surface topography modifications [69,74,75,76,77,78,79,80,81,82,83,84], these approaches are not the focus of this review due to the technical difficulties of adapting surface topological modifications to large-scale production. Further, the use of nanomaterials to enhance antibiofilm performance has been the subject of recent reviews by others [85].

**Figure 1:**
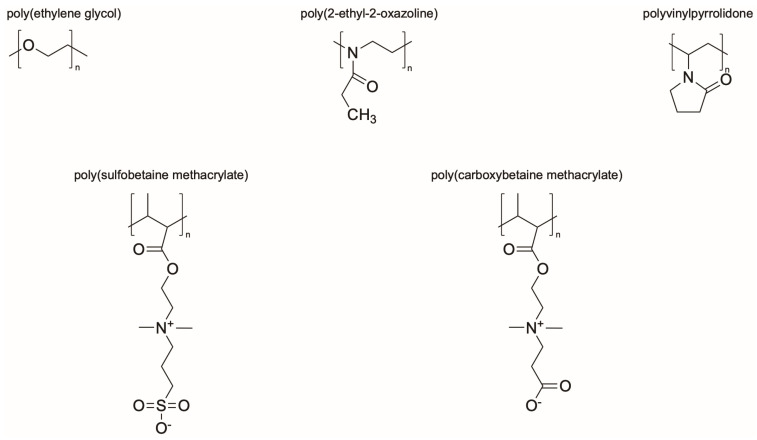
Representative structures of synthetic polymer coatings employed as nonfouling or bacteria-resistant coatings.

Synthetic polymer coatings are by nature more feasible for large-scale applications and their adaptation for biofilm prevention has made significant progress in recent years. Emerging synthetic polymer coatings believed to have significant antifouling capabilities include polyethylene glycol (PEG), polyvinylpyrrolidone (PVP), poly(oxazoline) (POZ), and zwitterionic polymers, as shown in Figure 1.

The hypothesized mechanism by which these hydrophilic polymers resist bacterial adhesion has evolved over the years as study into their properties has continued. The properties that have been observed as common among all of these polymers include being hydrophilic, electrically neutral, and a hydrogen bond acceptor but not a hydrogen bond donor [86,87]. Although these properties have been observed among these polymers, the main mechanism of resistance is theorized to be osmotic repulsion from the tightly bound water layers adjacent to the polymers [87,88,89,90]. In addition to this tightly bound water layer, it is important that the polymers have a net neutral charge. Any positively or negatively charged polymers, or localized regions of charge within a net neutral polymer, will absorb oppositely charged protein or bacteria via electrostatic interactions, leading to failure [87].

**Figure 2:**
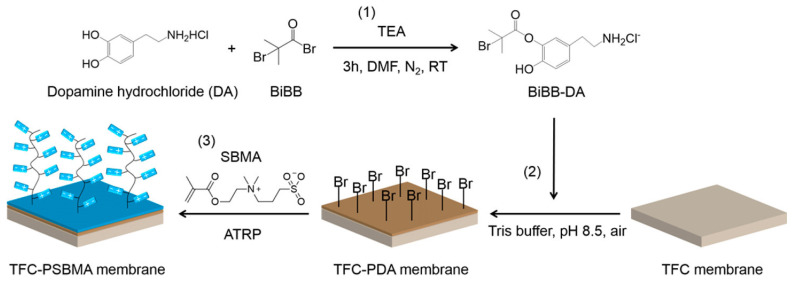
Coupling of initiator and dopamine hydrochloride (1), immobilization of initiators on the TFC membrane (2), and ATRP grafting of zwitterionic thin film (3). Reprinted with permission from [99]. Copyright 2017 American Chemical Society.

Prior to applying the bacteria-resistant polymer layer, most surfaces require modification to tightly anchor the polymer to the surface. Polymer brushes utilize surface-initiated atom transfer radical polymerization (ATRP), which commonly uses bromine-terminated surface-bound species [91]. Figure 2 is a schematic example of how the surface initiation process facilitates the formation of the subsequent polymer film using a dopamine-based surface-bound initiator. Other commonly used surface initiation techniques include silanes [92] or thiols [93,94,95], which contain the same terminal bromine-reactive group [96,97,98].

This review will focus on advances in these coatings over the last ten years. Two prior reviews by Banerjee et al. [46] and Yu et al. [100] effectively cover progress prior to 2015. Further, while many bacteria-resistant coatings have been combined with impregnated antimicrobial species, antimicrobials are not included in this review because of bacteria’s innate ability to develop resistance. Finally, polymers such as polyvinylpyrrolidone and poly (hydroxy functional acrylates) will not be discussed due to their limited investigation in the literature for bacteria prevention [101,102,103].

### 2.1. Polyethylene Glycol (PEG)

PEG has long been considered the gold standard for nonfouling coatings and is widely used in the manufacture of biofilm-resistant coatings. As such, it is also frequently used as the control standard in nonfouling experiments [97,104,105,106], which is why it is included in this review. While nonfouling polymers specifically refer to the ability to withstand exposure to 100% pure concentrations of plasma, serum, or blood with a nonspecific protein adsorption rate of less than 5 ng/cm^2^ [107], these polymers have also shown resistance to bacteria adhesion [108,109,110,111].

Several groups have reported demonstrations of the bacteria-resistant properties of PEG coatings on glass or silicon wafers using *Escherichia coli* (*E. coli*), *Staphylococcus aureus* (*S. aureus*), and *Pseudomonas aeruginosa* (*P. aeruginosa*). Additional commonly investigated bacteria discussed later in this review include *Staphylococcus epidermidis* (*S. epidermidis*), *Bacillus subtilis* (*B. subtilis*), and *Pseudomonas fluorescens* (*P. fluorescens*). *E. coli*, *P. aeruginosa*, and *P. fluorescens* represent Gram-negative strains, while *S. aureus*, *S. epidermidis*, and *B. subtilis* are Gram-positive. These bacteria are commonly selected due to their prevalence as the main pathogenetic infection sources in hospitals, biomedical implants, and water systems. Experimental findings, including the underlying substrate, the polymer coating process, the bacterial species, the experimental duration, and the results for PEG-based systems, are summarized in Table 1.

Dang et al. [96], Xing et al. [97], and Duanis-Assaf and Reches [98] all tested zwitterionic moieties alongside the PEG coatings and all three groups concluded that the zwitterionic coatings showed superior performances compared to the PEG coatings. These results are discussed in more detail below. Xing et al. [97] additionally found that the PEG coating exhibited fouling when tested with fluorescently labeled bovine serum albumin, whereas the zwitterionic coating did not. Buxadera-Palomero et al. [112] found that the pulsed electrodeposition had clearly superior, statistically different results compared to standard continuous electrodeposition for *S. aureus*. However, a significant decrease in *E. coli* adhesion was only observed for two of the five pulsed electrodeposition conditions.

Liu et al. [104] focused on the increasing grafting density of PEG, which has been shown to increase the subsequent desired bacterial adhesion resistance. Current methods of grafting from surfaces require reactions that make industrial applications unlikely [113], so the group investigated the efficacy of coatings produced by creating metal-polyphenol networks (MPNs) and attaching hexameric lysine PEG to the network (K6-PEG). Liu et al. reported that the procedures successfully increased grafting density to 4.06 chains/nm^2^, compared to previously reported grafting densities of only 0.79–1.9 chains/nm^2^ [106,114]. Grafting density is known to be directly related to nonfouling performance, so increasing grafting density in turn increases resistance to bacterial adhesion [113,115]. Liu et al. [104] reported a hundred-fold decrease in bacterial adhesion to the high-density PEG coating compared to the bare glass control.

Although there has been limited success using PEG coatings to reduce bacteria adhesion, this success is often dependent upon the underlying substrate. Many of the successful experiments utilize glass or silicon substrates, which unfortunately have limited applied uses. Stainless steel is a more practical substrate, but covering stainless steel with PEG coatings is ineffective in terms of resisting bacterial adhesion [116] and even further limited for preventing long-term biofilm formation [117]. Another shortcoming of the successful demonstrations of PEG coatings is their short experimental time frames, static experimental conditions, and lack of coating characterization. As shown in Table 1, most of the published studies are less than 24 h. These studies also rarely involve flowing bacteria species due to their short duration. Finally, PEG has shown susceptibility to autooxidation, especially in the presence of oxygen and transition metal ions, which is relevant for most applications [86,118,119,120]. Additional studies need to be pursued to demonstrate that PEG coatings resist bacterial adhesion on a wider range of substrates and for extended periods of time. With the limitations of PEG-based coatings, other chemistries are emerging with better ability to resist bacterial adhesion without having the same susceptibility to degradation.

### 2.2. Polyoxazoline (POZ)

**Figure 3:**
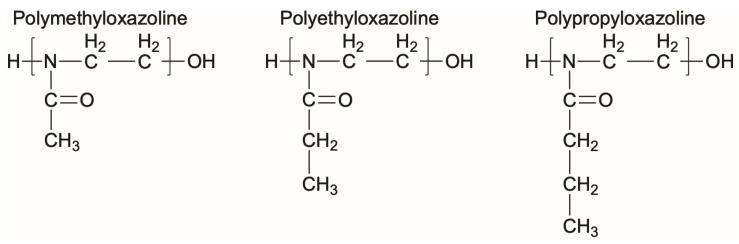
Representative chemical structures of three different polyoxazolines.

Polyoxazolines (POZs) are one family of chemistries that are not susceptible to the same oxidation that PEG suffers from [121], while also displaying effective resistance to bacteria adhesion. POZs are nonionic, stable, and have high solubilities in both water and organic solvents, making them well suited for many different applications [121]. In a similar timeline to PEG, POZs were first synthesized in the 1960s. However, their nonfouling or bacteria-resistant properties were not fully explored until the early 2000s due to their long reaction times and perceived limitations in terms of applications [122,123]. Recently, POZs have been employed for many biomedical applications including surface coatings that can control fouling and bacterial adhesion [122,123,124,125]. A few examples of the chemical structures of POZs are shown in Figure 3.

POZs have quickly risen in popularity and have become a frontrunner as bacteria-resistant polymer coatings. A recent review providing a significantly broader view on research into POZs was published by Arsenie and Lapinte [126]. A summary of key experimental results, specifically demonstrating POZ’s resistance to bacterial adhesion, is provided in Table 2, including the underlying substrate, the polymer coating process, the bacterial species, the experimental duration, and the results.

While these studies all demonstrate promise, the performance is not as good as that of PEG-based coatings and there are additional similar concerns to those raised for PEG-based coatings. For example, there is a large gap in research into bacterial adhesion over extended exposure times, as the longest study was only 24 h. Additionally, there are limited investigations under flowing conditions. Finally, there is an even greater lack of diversity of underlying substrates than was seen for PEG, and multiple studies lack quantification of bacterial adhesion and the coating physical characteristics. POZs show potential, but considerable additional advancements are necessary before they can be applied broadly.

### 2.3. Zwitterions and Polyampholytes

Zwitterionic hydrogels and coatings have emerged among the highest-performing subsets of nonfouling polymers. Zwitterionic refers to chemistries which contain an equal number of closely spaced cationic and anionic groups. Polyampholytes are a subset of zwitterions that combine co-localized anionic and cationic monomers to create a net neutral system that behaves similar to their zwitterionic analogs [134]. Zwitterionic polymers exhibit superior hydrophilicity to other polymers due to their large densities of anionic and cationic groups [135,136,137,138]. Additionally, electrostatic interactions allow for tunable control of desired mechanical properties [135,139]. Zwitterionic chemistries have also proven to not have susceptibility to oxidation degradation like PEG-based polymers [119,120]. The most common zwitterionic polymers include polyphosphorylcholine, polysulfobetaine, polycarboxybetaine, and polyampholyte chemistries, although others including pseudo-zwitterions do exist. The Jiang group, among others, has made significant advances, demonstrating bacterial adhesion-resistant zwitterionic coatings that predate the scope of this review, but which are worth noting [91,118,140,141,142]. There is little recently published work utilizing polyampholytes as bacteria-resistant coatings, but there is current research in progress [108,109]. Table 3 summarizes the most common cationic and anionic groups found within most zwitterionic monomers [135].

Only one study of polyphosphorylcholine since 2015 was located, excluding those that also incorporate bactericides. However, phosphorylcholine research dates to the 1990s and there are many articles that predate this review [135]. Qian et al. [143] applied polyphosphorylcholine coatings to polyurethane-based uretal stents using immersion approaches, followed by UV curing. These were then challenged for 24 h against *E. coli* and *S. aureus* to evaluate bacterial adhesion resistance. The resistance was found to be 92.16% and 99.14%, respectively, indicating a strong performance.

Zwitterionic betaines, including phosphobetaine, carboxybetaine, and sulfobetaine, have received some of the most significant research efforts. Table 4 provides a summary of investigations of zwitterionic polymers including the underlying substrate, the polymer coating process, the bacterial species, the experimental duration, and the results.

As with the chemistries discussed above, critical gaps in zwitterionic coating research are the lack of detailed coating characterizations and the lack of diversity in the substrates that have been coated. More specifically, there is a lack of evaluations of films applied to metals, even though these substrates are widespread in applications where bacteria adhesion is problematic. However, two recent studies utilizing a zwitterionic thin film applied to metal were completed. Chen et al. [154] used 316L stainless steel substrates and prepared them with a one-step simultaneous polymerization and co-deposition of dopamine and poly(sulfobetaine methacrylate) (PSB) to create a polydopamine (PDA)/PSB coating. *P. aeruginosa* and *B. subtilis* adhesion over 24-h were investigated and the group reported a 99% reduction in the adhesion density of both species compared to bare stainless steel [154]. Sae-ung et al. [147] tested the adhesion of *S. aureus* to copolymers of 2-methacryloyloxyethyl phosphorylcholine (MPC) and methacrylate-substituted dihydrolipoic acid (DHLA) (poly(MPC-DHLA)) coated onto titanium. Sae-ung reported a reduction in adhered bacteria and biofilm formation after 1, 2, and 7 days for the poly(MPC-DHLA) compared to the uncoated titanium standard, although this was not quantified.

Other gaps in the zwitterionic research include the limited number of investigations involving flowing conditions and the lack of long-term studies. Many bacterial adhesion studies involve only 24–48 h of exposure to bacteria, with the majority using time points under 24 h. One recent study completed 14-day investigations, excluding the in vivo study discussed in the following section, but no studies beyond 14 days were discovered. Liu et al. [145] investigated bacterial adhesion to amino acid-based zwitterionic polymers at time points up to 14 days. The polymer brushes studied were composed of the amino acid-based monomers listed in Table 5 and results were compared to those obtained using a PEG coating.

**Figure 4:**
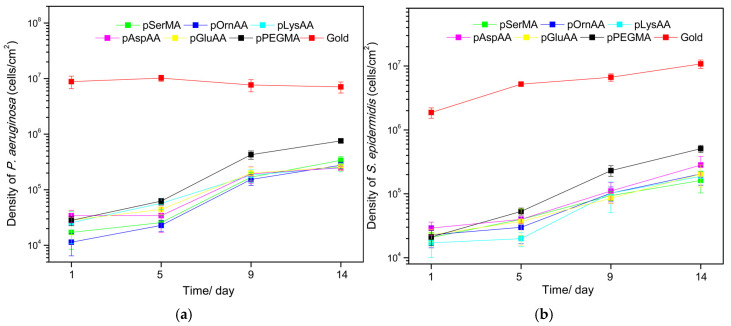
(**a**) Density of *P. aeruginosa* cells observed in cells/cm^2^ at time points of 1, 5, 9, and 14 days. (**b**) Density of *S. epidermidis* cells observed in cells/cm^2^ at time points of 1, 5, 9, and 14 days. Reprinted with permission from [145]. Copyright 2016 American Chemical Society.

Polymer brushes were applied to gold surfaces and exposed to either *S. epidermidis* or *P. aeruginosa* through a parallel flow chamber system. Samples were assessed for bacterial coverage and biofilm formation at time points of 1, 5, 9, and 14 days. After one day, the PEG and zwitterionic coatings had similar coverage and resistance to bacteria. However, by 14 days, the PEG coating displayed more bacteria than the zwitterionic coating. The results observed are reproduced with permission below in Figure 4a,b. Figure 4a shows the density of *P. aeruginosa* cells observed and Figure 4b shows the density of *S. epidermidis* cells observed per square cm.

In Figure 4a,b, it is obvious that at the longer time points all of the polymers’ performances decrease. At the 14-day time point, the group reported that the PEG coating had 10.7% and 4.7% surface coverage accumulations of *P. aeruginosa* and *S. epidermidis*, respectively. These values were significantly greater than those of the zwitterionic coatings, which all had less than 2.6% surface coverage for both bacteria species. These results also highlight the need for studies beyond the 24–48-h time points given the increases in bacterial surface coverage over time.

A third gap, not identified earlier, but applicable to all of the chemistries covered in this review, is the significant lack of in vivo studies involving bacteria. Shen et al. [156] tested zwitterionic coatings, both in vitro and in vivo, against *S. aureus* and *S. epidermidis*. The group used a photo-grafting technique, which simultaneously polymerized sulfobetaine methacrylate (SBMA) or carboxybetaine methacrylate (CBMA) with a PEG crosslinker onto a poly(dimethyl) siloxane (PDMS) substrate. 2-Hydroxyethyl methacrylate (pHEMA) was used as the non-zwitterionic control coating.

**Figure 5:**
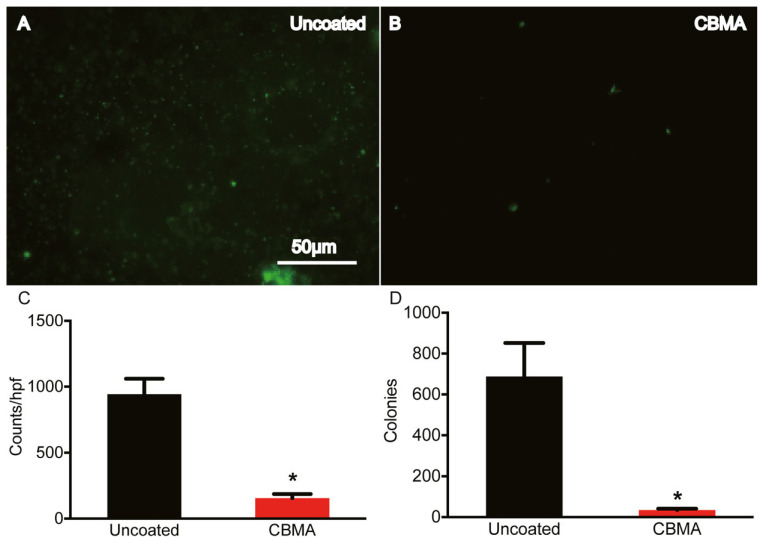
Microscopic images with analysis on uncoated (**A**) and CBMA coated (**B**) implants at 21 days with quantitative analysis in (**C**,**D**). The scale bar in (**A**) is also representative of (**B**). * indicates statistically significant results (*p* < 0.0001). Reprinted with permission from [156]. Copyright 2021 American Chemical Society.

Two in vitro tests were conducted. The first immersed the coated samples into a 10^8^ bacterial suspension (wet) and the second sprayed the suspension onto the substrate to imitate inoculation of an implant (droplet). For both conditions, samples were incubated for periods of both 24 and 48 h. It was found that there was a statistically significant decrease in both *S. aureus* and *S. epidermidis* adhesion to the CBMA polymer compared to the controls for both tests. However, the SBMA polymer only showed a statistically significant reduction in *S. aureus* adhesion, but not for *S. epidermidis*, under both wet and droplet conditions. Because the CBMA coatings were more reliable at preventing *S. aureus* and *S. epidermidis* adhesion compared to the SBMA coatings, they were the only coatings evaluated in vivo. The in vivo test consisted of inoculating the implant with *S. aureus* at the site of implantation. After 21 days, the samples were explanted and it was found that there was a statistically significant reduction in *S. aureus* compared to uncoated implants. This can be seen in Figure 5 [156].

While all of these studies reported successes, it is also worth noting that the best results were typically reported with sulfobetaine-based coatings. Table 4 shows that each experiment that reported a reduction in adhesion of over 98% was for thin films composed of a sulfobetaine monomer. This could, in part, be attributed to its good chemical stability and less sensitive pH-dependent properties than other zwitterionic betaine species [135,157,158,159,160]. However, the in vivo results contradict this, suggesting that further investigations are still necessary. Further complicating our ability to directly compare the results obtained in different studies to identify the highest-performing chemistry is the lack of consistent experimental parameters. Across the sixteen studies summarized in Table 4, there are fifteen different coated substrates evaluated with six different bacteria strains over thirteen varying time points. As such, side-by-side comparisons between different chemistries are not possible unless they are directly compared within a study.

## 3. Conclusions

There have been many advancements in the use of polymer coatings to resist bacteria adhesion. In particular, zwitterionic coatings have demonstrated the strongest capacity to prevent bacterial adhesion across the widest variety of bacteria species, especially sulfobetaine-based systems. However, despite the significant advancements, there are still systematic shortages of long-term studies, evaluations under flowing conditions, detailed characterizations of the coating properties, and assessments for a diversity of underlying substrate compositions. To successfully address these shortcomings, different mechanical properties, film thickness, and coating approaches may be necessary. However, these variables must also be balanced with the performance requirements for the intended industrial and biomedical applications. The development and demonstration of techniques capable of coating large-scale systems is also necessary.

## Figures and Tables

**Table 1:** Summary of recent investigations into bacterial resistance of PEG coatings.

Author	Substrate	Coating Application	Bacteria Species	Time	Efficacy
Dang et al. [96]	SPR chips, glass	Immersion, drop coating, spincoating	*E. coli*, *S. aureus*, *P. aeruginosa*	1-day 3-days 7-days	99% suppression
Xing et al. [97]	Silicon wafers	Drop coating	*E. coli*, *S. aureus*, *P. aeruginosa*	7-days	99% reduction
Buxadera-Palomero et al. [112]	Titanium	Pulsed electrodeposition	*E. coli*, *S. aureus*	2-h	up to 90% reduction
Duanis-Assaf and Reches [98]	Glass	Polymer brush	*E. coli*	30 min	~80% maximum reduction in adhesion
Liu et al. [104]	Glass	Immersion	*E. coli*, *S. aureus*	8-h	99% reduction

**Table 2:** Summary of recent investigations into bacterial resistance of POZ coatings.

Author	Substrate	Coating Application	Bacteria Species	Time	Efficacy
Cavallaro et al. [127]	Glass	Plasma deposition	*S. epidermidis*	24 h	>89.8% reduction
Ramiasa et al. [128]	Tissue culture plate	Plasma deposition	*S. epidermidis*	24 h	Biofilm not well adhered *
Al-Bataineh et al. [129]	Silicon wafers	Plasma deposition	*S. epidermidis*	24 h	Maximum 80% reduction at center of sample
He et al. [130]	Silicon wafers	Spincoating	*E. coli* ^1^ *S. aureus* ^2^	1 h	Max 0.9% reduction ^1^ Max 0.3% reduction ^2^
He et al. [131]	Silicon wafers	Layer by layer immersion deposition	*E. coli*, *S. aureus*	1 h	Maximum reduction of ~90%
Li et al. [132]	Silicon wafer, glass	Layer by layer immersion deposition	*E. coli*, *B. subtilis*	6 h	Reduced adhesion by 98%
Portier et al. [133]	Silicon wafers	Bar coating	*S. aureus*, *P. aeruginosa*, *Pseudoalteromonas*	2 h	Adhesion strength reduced, fouling release increased *

* indicates that bacterial adhesion was not reported as quantified data. ^1^ or ^2^ denotes the specific bacterial species with the corresponding results.

**Table 3:** Common cationic and anionic substituents found within zwitterions. This table was reproduced under an Elsevier Creative Commons license from [135].

Types of Charged Groups	Structures of Charged Groups		
Cationic groups	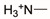	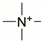	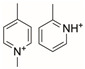
	Amino	Quaternary ammonium	Pyridine
Anionic groups	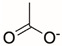	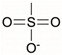	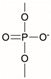
	Carboxylate	Sulfonate	Phosphate

**Table 4:** Summary of recent investigations into the bacterial resistance of zwitterionic coatings including carboxybetaine (CB), sulfobetaine (SB), and phosphobetaine (PB) coatings.

Author	Substrate	Coating Application	Bacteria Species	Time	Efficacy
Liu et al. [99]	Composite membrane	SB polymer brush	*E. coli*	3 h	90% reduction in CFU
Hassani et al. [144]	Silicone rubber	CB polymer brush	*S. aureus*, *E. coli*	24 h	Significant reduction to bare silicone *
Liu et al. [145]	Gold	Amino acid-based polymer brush	*S. epidermidis P. aeruginosa*	1, 5, 9, 14 days	>97% maximum reduction
Shafi et al. [2]	Koch membrane	SB chemical vapor deposition	*P. aeruginosa*	2 h	99.6% reduction in adhesion
Khlyustova et al. [92]	Glass (PVC control)	SB chemical vapor deposition	*P. aeruginosa * *B. subtilis*	24 h	87% reduction 75% reduction
Karthäuser et al. [146]	Glass	SB spincoating	*E. coli*, *B. subtilis*, *P. fluorescens*	45 min	98% maximum reduction
Sae-ung et al. [147]	Titanium	Phosphorylcholine spincoating	*S. aureus*	1, 2, 7 days	Significant reduction *
Venault et al. [148]	None	PB solution casting	*E. coli*	3 h, 24 h	>90% reduction
Yin et al. [149]	None	SB solution casting	*S. aureus* ^1^ *E. coli* ^2^	30 min	94.15% reduction ^1^ 94.27% reduction ^2^
Wang et al. [150]	None	CB solution casting	*S. aureus*	1 h	Minimal adhesion observed *
Cao et al. [151]	Silicone rubber	SB covalent grafting	*S. aureus* ^1^ *E. coli* ^2^	3 h	82.1% max reduction ^1^ 74.2% max reduction ^2^
Texidó et al. [152]	Polydimethyl siloxane	SB immersion	*E. coli*	24 h	99% reduction
Ran et al. [153]	Glass, silicon wafers	SB immersion	*E. coli*	4 h ^1^ 24 h ^2^	Lowest adhesion rate 5% ^1^; Lowest adhesion rate 7% ^2^
Chen et al. [154]	Stainless steel	SB immersion	*P. aeruginosa*, *B. subtilis*	24 h	99% reduction
Venault et al. [155]	PVDF membranes	SB bath procedure	*E. coli*	3 h	100% maximum reduction
Shen et al. [156]	PDMS	CB, SB photo-grafting	*S. aureus*, *S. epidermidis*	21 days	Reductions of >500 counts/hpf

* indicates that bacterial adhesion was not reported as quantified data. ^1^ or ^2^ denotes the specific bacterial species with the corresponding results.

**Table 5:** Chemical structures of amino acid zwitterionic monomers. Reprinted with permission from [145]. Copyright 2016 American Chemical Society.

Full Name of Monomer	Abbreviated Name	Chemical Structure
Serine Methacrylate	SerMA	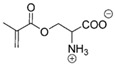
Ornithine Methacrylamide	OrnAA	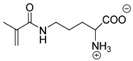
Lysine Methacrylamide	LysAA	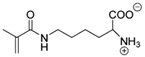
N^4^-(2-methacrylamidoethyl) asparagine	AspAA	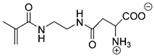
N^5^-(2-methacrylamidoethyl) glutamine	GluAA	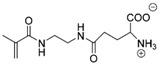

## Data Availability

No new data were created or analyzed in this study. Data sharing is not applicable to this article.
